# Bone Morphogenetic Protein-2 Conjugated to Quantum Dot^®^s is Biologically Functional

**DOI:** 10.3390/nano10061208

**Published:** 2020-06-20

**Authors:** Daniel Halloran, Vrathasha Vrathasha, Hilary W. Durbano, Anja Nohe

**Affiliations:** Department of Biological Sciences, University of Delaware, Newark, DE 19716, USA; dhallor@udel.edu (D.H.); vrathash@udel.edu (V.V.); weidnerh@udel.edu (H.W.D.)

**Keywords:** BMP-2, FTIR, Bone: QDot^®^s, fluorescence imaging, mineralization

## Abstract

Quantum Dot^®^s (QDot^®^s) are novel, semi-conductive nanostructures that emit a certain fluorescence when excited by specific wavelengths. QDot^®^s are more photostable, brighter, and photobleach less than other fluorescent dyes. These characteristics give them the potential to be used in many biological applications. The shells of QDot^®^s are coated with functional groups, such as carboxylate and organic groups, allowing them to couple to peptides/proteins and be used for real-time imaging and high-resolution microscopy. Here, we utilize Quantum Dot^®^s and Bone Morphogenetic Protein-2 (BMP-2) to create a BMP-2-QDot^®^s conjugate. BMP-2 is a growth factor that drives many processes such as cardiogenesis, neural growth, and osteogenesis. Despite its numerous roles, the trafficking and uptake of BMP-2 into cells is not well-established, especially during progression of diseases. The results presented here demonstrate for the first time a fluorescent BMP-2 analog that binds to the BMP-receptors (BMPRs), remains biologically active, and is stable for long time periods. Previous attempts to develop a biological BMP-2 analog with Fluorescein isothiocyanate (FITC) or nanodiamonds lacked data on the analog’s stability. Furthermore, these analogs did not address whether they can signal within the cell by binding to the BMPRs or were mediated by non-stable conjugates.

## 1. Introduction

Quantum Dots (QDot^®^s) are novel, semi-conductive nanomaterials used for many biological applications such as tumor targeting, cellular environment imaging, and cancer metastasis tracking [1,2,3,4,5,6,7]. Experimental usage of QDot^®^s has surpassed traditional dyes such as green fluorescent proteins (GFPs) and fluorescein isothiocyanate (FITC) due to their superior photostability, brightness, and small particle size [8,9,10]. Furthermore, the outer shell of QDot^®^s can be modified for its intended purpose; for example, this shell can be carboxylated to allow for coupling between QDot^®^s and amine groups of proteins or peptides [11,12]. These characteristics of QDot^®^s along with its capability to attach to proteins allow for real-time imaging, high resolution microscopy, and in vivo experimentation. 

Utilization of QDot^®^s provides the opportunity to elucidate mechanisms of proteins that may be implicated in diseases such as cancer, osteoporosis, and osteosarcoma. Further to this, proteins that are well-studied but lack research on their trafficking and uptake can be identified by using QDot^®^s, such as when these proteins are involved in aberrant signaling and disease progression. A specific example is bone morphogenetic protein-2 (BMP-2), which is implicated in aberrant signaling during osteopenia and osteoporosis, however, the mechanism of this pathway is unknown [13,14,15,16]. By conjugating QDot^®^s to BMP-2, the altered signaling pathways can be determined.

BMP-2 is a member of the largest subgroup of the transforming growth factor-β (TGF-β) superfamily and has crucial roles in development, cardiogenesis, neurogenesis, adipogenesis, chondrogenesis, and osteogenesis [17,18,19,20,21,22,23,24,25,26]. Furthermore, BMP-2 is a critical factor that commits mesenchymal stem cells (MSCs) to differentiate into osteoblasts, which are responsible for forming new bone [12,18,27]. Due to its multifunctionality, the Food and Drug Administration (FDA) approved usage of recombinant human BMP-2 (rhBMP-2) during anterior lumbar interbody spinal fusion (ALIFs) surgeries in 2002 [28,29,30,31,32,33]. 

BMP-2 functions by activating downstream signaling pathways. First, BMP-2 is secreted and binds to bone morphogenetic protein receptors (BMPRs), including BMPRIa, BMPRIb, and BMPRII, which are serine/threonine kinases [13,25,34,35,36,37,38]. Most commonly, BMP-2 will bind to BMPRIa and BMPRII complexes. These complexes can localize in clathrin coated pits (CCPs), caveolae, or lipid rafts [34,37,39,40,41]. Once the protein binds to BMPRII, casein kinase 2 (CK2) is released from BMPRIa, which exposes a glycine-serine (GS) rich homeobox domain. BMPRII phosphorylates this GS box, leading to activation of Smad (canonical) and non-Smad (non-canonical) signaling pathways [42,43,44]. BMPRs localized in caveolae will lead to activation of Smad signaling, while BMPRs localized in CCPs will activate non-Smad signaling [34,37,39,45]. In the Smad pathway, BMPRIa phosphorylates Smad 1, 5, and 8, which recruit Smad4. The complex then translocates to the nucleus to serve as a transcription factor for genes such as *RUNX2*, which is a master regulator of other genes such as *Osterix* [17,20,35]. In the non-Smad pathway, BMPRIa activates pathways such MAPK, ERK, and PI3K to regulate cell survivability and proliferation [15,46]. 

Despite the ability of BMP-2 to activate multiple pathways, the association of BMP-2 with BMPRIa and the shuttling or endocytosis of the complexes have not been fluorescently labeled. Previously, peptide-derived BMP-2 was conjugated to oxidized detonation nanodiamonds, however, its functionality or fluorescent activity remains unknown [47]. Additionally, BMP-2 was bound to a FITC fluorescent dye, however, the stability and functionality of this conjugation was not assessed as FITC bleaches rapidly [45]. To elucidate mechanisms of BMP-2 signaling, we use QDot^®^s. In our study, the QDot^®^s are carboxylated to attach to the lysine amino acids of BMP-2 using N,N’-Dicyclohexylcrbodiimide (DCC) as the coupling reagent. Vrathasha et al. 2018 demonstrated that the peptide CK2.3, which inhibits activity of casein kinase 2 (CK2), can be derivatized at the lysine residue to form an amide bond with the QDot^®^s [1]. Here, we demonstrate a functionally active and stable conjugation between BMP-2-QDot^®^s for at least 14 days. Additionally, the BMP-2-QDot^®^s conjugation colocalizes with BMPRs by 1 h, and the conjugation increases mineralization similar to unconjugated BMP-2. This is the first study in which the stability and functionality of a BMP-2 probe was assessed, and this advancement will allow us to elucidate more functions of BMP-2 during the progression of diseases.

## 2. Materials and Methods 

### 2.1. Conjugation of BMP-2 to QDot^®^s

To conjugate BMP-2 to QDot^®^s, we used the following method. For 30 min in the dark, 10 μL of 40 nM recombinant BMP-2 (GenScript, Piscataway, NJ, USA) was placed in a solution with 2 μL of 8 μM QDot^®^s (QDot^®^525 ITK carboxyl quantum dots, catalog #Q21341MP, Invitrogen, Carlsbad, CA, USA) and the proceeding reagents: 84 μL of dimethyl sulfoxide (DMSO, Fisher Scientific, Pittsburg, PA, USA), 2 μL of DCC (36 mg in 1 mL of DMSO, Sigma-Aldrich, St. Louis, MO, USA), and 2 μL of 10X phosphate buffered saline (PBS). After, 200 μL of 1X PBS was added to quench the reaction and put on ice, for a total volume of 300 μL. To verify successful conjugation, five different combinations of the reagents listed above were used as controls, which were 1X PBS only, DCC only, QDot^®^s only, QDot^®^s and PBS, and BMP-2-QDot^®^s and QDot^®^s without DCC. 

### 2.2. Size Exclusion Chromatography (SEC)

To separate the conjugation from other reagents, SEC was performed. Medium Sephadex beads (Sigma-Aldrich, St. Louis, MO, USA) were purchased and suspended in diH2O overnight. Next, 3 mL of the beads were packed into columns and centrifuged at 2000 rpm for 5 min. Slowly, the conjugation and control solutions were added to the columns, 100 μL at a time. Once added, 100 μL of diH2O was added dropwise to each column. Each 100 μL that passed through the columns was considered a fraction, and these fractions were collected in microcentrifuge tubes. These fractions were then analyzed using UV/VIS and FTIR spectroscopy.

### 2.3. UV/VIS Spectroscopy

After drop-casting 2 μL of the collected fractions 3X on the pedestal of the NanoDrop^®^ Spectrophotometer, UV/VIS spectra were collected to confirm the conjugation between BMP-2 and QDot^®^s. These spectra were gathered by plotting the absorbance of the sample with a range of wavelengths (220–300 nm). Then, a standard curve of QDot^®^s was created to determine the concentration of the conjugation in the fractions. All conjugate concentrations were normalized to 40 nM, which was used to stimulate C2C12 cells in subsequent experiments. 

### 2.4. Fourier Transform Infrared (FTIR) Spectroscopy 

FTIR was used to determine the success and stability of BMP-2-QDot^®^s conjugation. FTIR collects mid-infrared spectra by measuring reflectance. For measurement, the conjugation fraction was added to gold-coated rounded coverslips (Sigma-Aldrich, St. Louis, MO, USA) by adding 10 μL three times and desiccating the sample. The dried sample was analyzed using Bruker Optics and a Hyperion 2000 microscope with a Mercury Cadmium Telluride (MCT) detector. The spectra included 64 scans with a resolution of 4 cm^−1^. To acquire the spectra, OPUS v6.0 was used and the spectra were corrected and normalized using the FTIR software. This experiment was repeated independently at least three times. 

### 2.5. Cell Culture

C2C12 (murine myoblast derived) cells were purchased from American Type Culture Collection (Manassas, VA, USA) and used for all experiments. The cells were grown in Dulbecco’s Modified Eagle’s Medium (DMEM, Hy-clone, Pittsburgh, PA, USA), supplemented with 10% Fetal Bovine Serum (FBS, Gemini Bio-products, West Sacramento, CA, USA), 1% penicillin/streptomycin (Hy-clone, Pittsburgh, PA, USA), and 1% anti/antifungal (Hy-clone, Pittsburgh, PA, USA). Once reaching confluency, cells were serum starved overnight in DMEM without FBS. The cells were left unstimulated, treated with BMP-2 or BMP-2-QDot^®^s. 

### 2.6. Immunofluorescent Labeling

C2C12 cells were grown on coverslips in 12-well plates for three days after being plated with a cell density of 1 × 10^4^. The cells were grown in DMEM with 10% FBS and at the end of the third day, the cells were serum starved overnight by being placed in DMEM without FBS. On the next day, the cells were stimulated with 40 nM BMP-2-QDot^®^s or left unstimulated for 1 h. After stimulation, the cells were fixed with 4.4% paraformaldehyde (PFA, pH 7.2, Sigma-Aldrich, Pittsburgh, PA, USA) for 15 min. The cells were washed 3 times with 1X ice-cold PBS (pH 7.4) and permeabilized with 0.01% saponin (Sigma-Aldrich, St. Louis, MO, USA) diluted in 1X PBS for 10 min on ice. The cells were then incubated for 1h on ice with 3% bovine serum albumin (BSA, Fisher Scientific, Pittsburgh, PA, USA) dissolved in 1X PBS to block non-specific binding. The cells were labeled with rabbit polyclonal anti-BMPRIa primary antibody (Catalog #sc-134285, Santa Cruz, Dallas, TX, USA) diluted in 1% BSA with a ratio of 1:1000 for one hour. The cells were washed with 1X PBS, and then, incubated with a fluorescently tagged donkey-anti-rabbit IgG H&L secondary antibody (Alexa Fluor^®^633, Catalog #A21052, Fisher Scientific, Pittsburg, PA, USA) diluted in 1% BSA with a ratio of 1:1000 for one hour. Nuclei of the cells were then stained using 100 μL (0.5 ng/mL) of Hoechst (Sigma-Aldrich, St. Louis, MO, USA) for 2.5 min. The coverslips were attached to glass slides using Cytoseal™ (Thermo Scientific, Waltham, MA, USA). Slides were then imaged using Zeiss LSM710 at 63×/1.4 Plan-Apochromat oil objective (Delaware Biotechnical Institute, Newark, DE, USA). 

### 2.7. Von Kossa Assay

C2C12 cells were grown in 24-well plates for two days after being plated with a cell density of 1 × 10^4^. The cells were grown in DMEM with 10% FBS and after the end of the second day, the cells were placed in DMEM without FBS overnight. The next day, the cells were then left unstimulated, stimulated with 40 nM BMP-2, or stimulated with 40 nM BMP-2-QDot^®^s. Two days later, the cells were supplemented with DMEM with 10% FBS and the next day, the cells were stimulated again. On day five, the cells were washed 3 times with ice-cold PBS and fixed with 4.4% PFA for 15 min. The cells were washed 3 times with 1X PBS and incubated with 5% silver nitrate (Chem-Impex International, Wood Dale, IL, USA) dissolved in diH2O and exposed to UV light for 1 h. The cells were then washed 3 times with diH2O and 5 times with 1X PBS and allowed to dry for two days. The cells were imaged with the Zeiss Axiovert 10 microscope at 5×/12 Achrostigmat objective; 15 random images were taken of each well. The images were then analyzed with ImageJ (NIH, Bethesda, MD, USA), converted to 8-bit, and the threshold was set to 80. Mineralization deposits were measured using the ‘analyze particles’ function. Outliers were removed by using the Chauvenet’s criterion test. Data obtained were analyzed using the single factor analysis of variance (ANOVA) and statistical analysis was performed by using the Student’s *t*-test. This experiment was repeated independently at least three times. 

## 3. Results

### 3.1. Development of the BMP-2-QDot^®^s Fluorescent Probe

**Figure 1:**
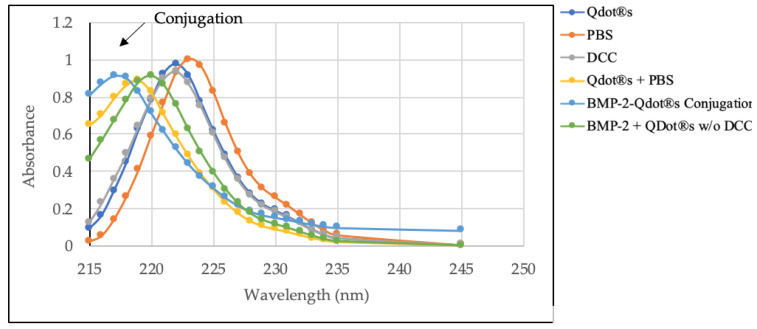
UV/VIS spectra of BMP-2-QDot^®^s and five control fractions between wavelength 215–245 nm. BMP-2-QDot^®^s was shifted left of the control samples, due to the absorbance of secondary amides.

Here, we are interested in creating a BMP-2 analog that allows us to determine the trafficking of BMP-2. In order to form a bond between BMP-2 and QDot^®^s, the QDot^®^s were carboxylated to form an amide bond with BMP-2. To form this bond between BMP-2 and the QDot^®^s, we utilized DCC as a coupling agent to serve as a hydrogen donor to form the amide bond. This conjugation was followed by SEC to separate the conjugation from non-specific reactants. Using these methods, our results demonstrate that BMP-2 was conjugated to QDot^®^s in the presence of DCC. The fractions that eluted from the columns in SEC were analyzed using UV/VIS spectroscopy to verify the conjugation. The spectra analyzed included the conjugated fraction along with five control fractions. The spectra of BMP-2-QDot^®^s displayed a peak that was shifted from the other controls, between the wavelengths of 220–225 nm (Figure 1). In total, 25 fractions were collected, and the reactants not included in the conjugation fraction were eluted in other fractions. Data were normalized to PBS as the control. 

### 3.2. Stability of the BMP-2-QDot^®^s Fluorescent Probe

**Figure 2:**
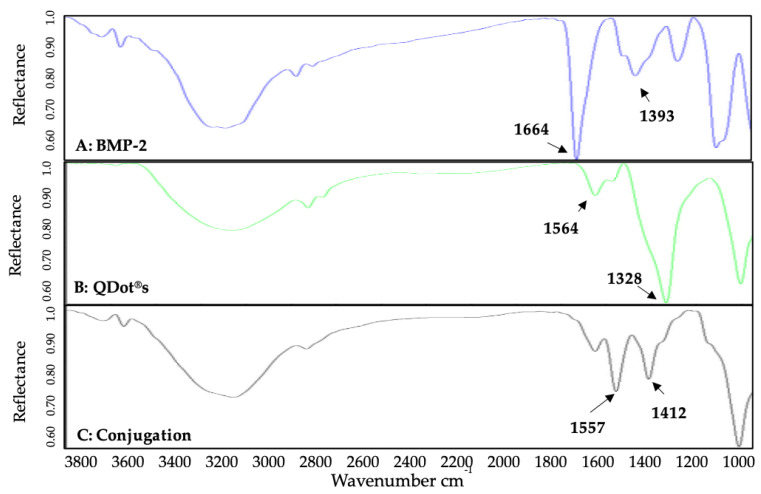
FTIR spectrometer readings of BMP-2 (**A**), QDot^®^s (**B**), and BMP-2-QDot^®^ (**C**) samples drop-casted onto gold-coated coverslips. Samples were collected using Bruker Optics and a Hyperion 2000 microscope with a Mercury Cadmium Telluride (MCT) detector with 64 scans at a resolution of 4 cm^−1^. Experiments were repeated at least three times.

**Figure 3:**
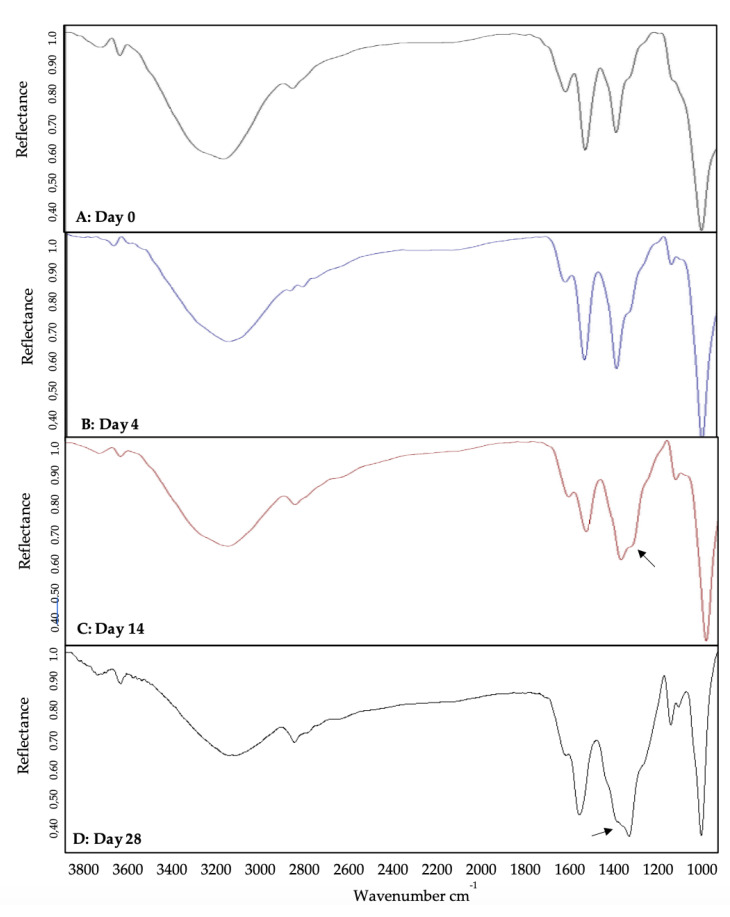
BMP-2-QDot^®^s stability analyzed using FTIR spectroscopy. On day 0 and day 4, the BMP-2-QDot^®^s conjugate is still stable (**A**,**B**). However, by days 14 and 28, the conjugation is falling apart as depicted by curve shifts and shoulder formation indicated by the arrow (**C**,**D**).

To determine the bond-specific attachment of BMP-2 to QDot^®^s, we utilized FTIR. FTIR is an instrument used to obtain a specific wavelength that would display amide bond peaks to indicate the conjugation. Further to this, we utilized FTIR over 28 days to elucidate the conjugate’s stability. Initially, BMP-2 only, QDot^®^s only, and the BMP-2-QDot^®^s probe were analyzed via FTIR. For the BMP-2 spectrum (Figure 2A), peaks were identified at 1664 and 1393 cm^−1^, representing two different amide bonds [48,49]. In the QDot^®^s only spectrum (Figure 2B), peaks were identified at both 1564 and 1328 cm^−1^, representing carboxylate peaks [50]. Finally, the BMP-2-QDot^®^s spectrum (Figure 2C) contained peaks at 1557 and 1412 cm^−1^. These peaks indicate a successful conjugation because when BMP-2 is bound to the QDot^®^s, a resonance effect is produced, moving the peaks to a lower frequency [51,52,53]. To determine the stability of the BMP-2-QDot^®^s probe, FTIR was used to analyze the conjugation samples for 28 days. For 0 and 4 days, the probe appeared to be stable (Figure 3A,B). However, at 14 and 28 days, a shoulder is formed, and the peaks have shifted to 1580 and 1343 cm^−1^, indicating that BMP-2 has been detached from the QDot^®^s (Figure 3C,D). 

### 3.3. Colocalization of BMP-2-QDot^®^s and BMPRIa

**Figure 4:**
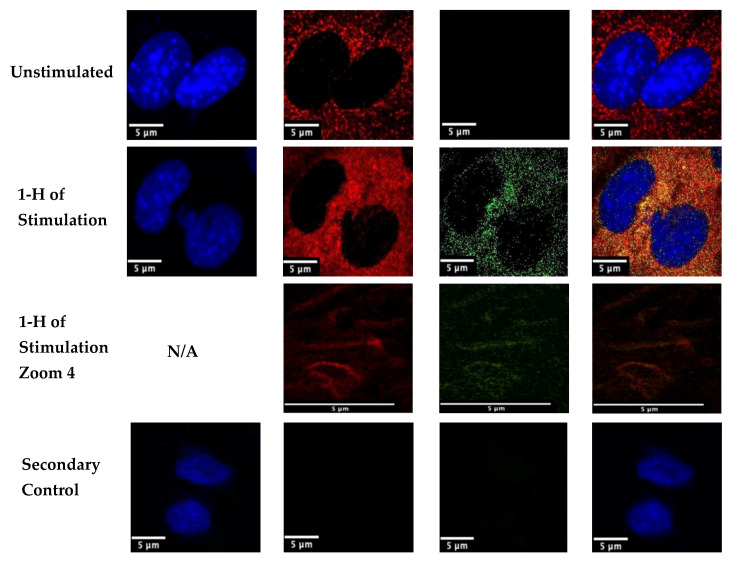
Stimulation of C2C12 cells with BMP-2-QDot^®^s for 1 h. Cells were plated at a density of 1 × 10^4^ per well and were stimulated or left unstimulated. After stimulation, cells were fixed with 4.4% PFA and BMPRIa (red) and the nuclei (blue) were stained. The nucleus is missing from the ‘1 h of Stimulation Zoom 4’ as the image is taken of the plasma membrane. Confocal microscopic images were taken using the Zeiss LSM710 with the 63×/1.4 objective to determine colocalization (yellow) between BMP-2-QDot^®^s and BMPRIa. Each image includes one or two representative cells for each treatment condition. Arrows indicate colocalization. This experiment was repeated independently at least two times.

BMP-2 is a growth factor that binds to type I and type II Bone Morphogenetic Protein Receptors (BMPRs). When active, BMP-2 binds to these receptors to initiate several downstream signaling cascades. Therefore, we utilized confocal microscopy to determine if the BMP-2 analog was able to colocalize with its receptors, BMPRIa and BMPRII. Previous studies utilized FITC to observe uptake of BMP-2, but not the colocalization of BMP-2 with its receptors. Here, we stimulated C2C12 cells for 1h with the 40 nM BMP-2-QDot^®^s conjugation or left them unstimulated. Then, using immunofluorescent labeling and confocal microscopy, we determined that BMP-2-QDot^®^s were able to colocalize with BMPRIa (yellow color), demonstrating that this BMP-2 analog retains its biological activity. Furthermore, in the 1 h Stimulation Zoom 4, we demonstrate the BMP-2-QDot^®^s conjugation completely colocalizes with BMPRIa in the plasma membrane (Figure 4).

### 3.4. Functionality of the BMP-2-QDot^®^s Probe

**Figure 5:**
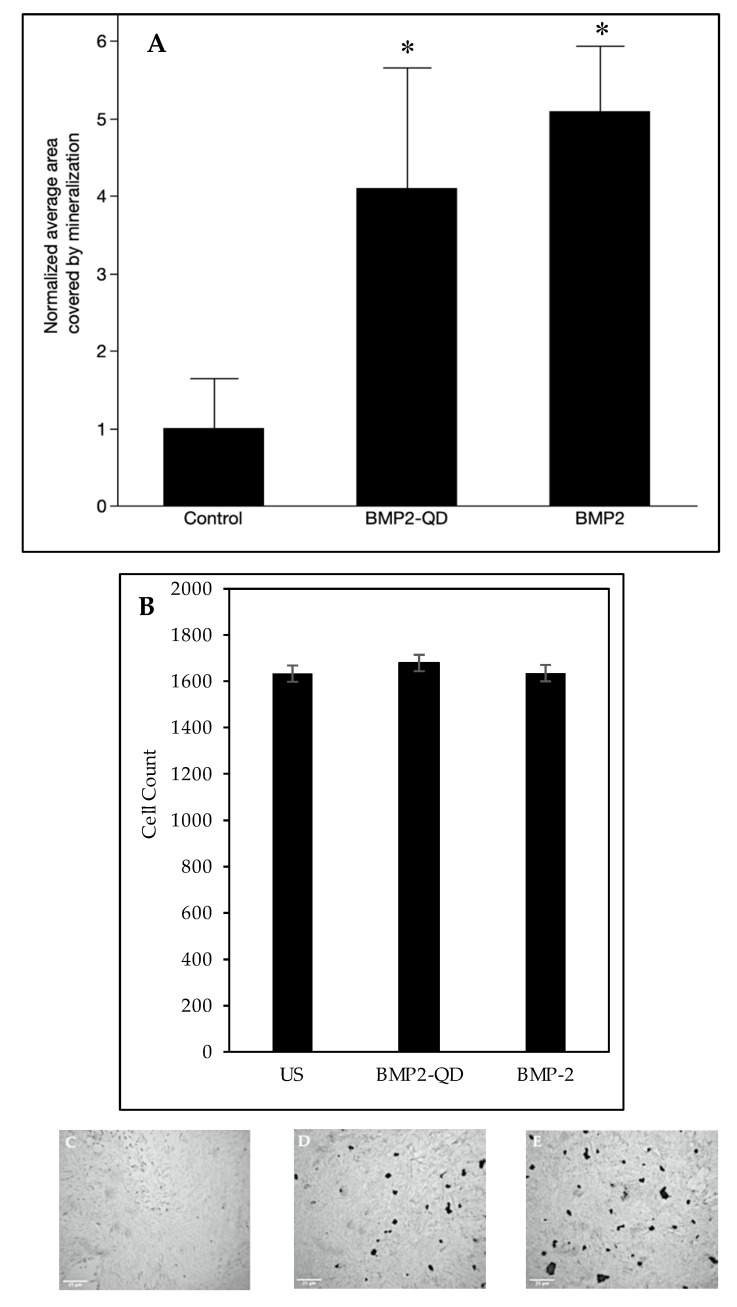
(**A**) Mineralization deposits of C2C12 cells left unstimulated, treated with 40 nM BMP-2, or treated with 40 nM BMP-2-QDot^®^s was determined using a von Kossa assay after five days. Cells were plated at a density of 1 × 10^4^ cells per well. In total, 15 Images were randomly obtained for each condition using the Zeiss Axiovert 10 microscope at 5×/12 Achrostigmat objective. (**B**) Cell count to demonstrate viability of cells. At least five images were counted using ImageJ following the von Kossa assay. (**C–E)** Representative images of the cells from each treatment are located underneath the bar graph. This experiment was repeated independently at least three times. Data were analyzed using ImageJ and one-way ANOVA followed by the Student’s *t*-test. * indicates statistical significance at *p* < 0.05 when compared to unstimulated cells.

BMP-2 activates downstream signaling pathways that lead to an induction of bone mineralization. Previously, BMP-2 was able to induce mineralization in C2C12 cells in a von Kossa assay, which is a method to visualize phosphate and calcium deposits. Here, we used this same assay to determine if our BMP-2 analog was functional. C2C12s were treated with BMP-2-QDot^®^s, treated with BMP-2 only, or left unstimulated. We demonstrated that BMP-2-QDot^®^s induced mineralization similar to BMP-2 only, indicating that our analog remains functional. Furthermore, both BMP-2-QDot^®^s and BMP-2 increased mineralization significantly higher than unstimulated cells (Figure 5). 

## 4. Discussion

For the first time, we report the stability and functionality of a BMP-2 analog. BMP-2 was conjugated to QDot^®^s because QDot^®^s are photostable, excitable, and limit photobleaching [7,9]. These qualities of QDot^®^s are optimal to observe protein colocalization and movement [3,4,7,10,12]. Further to this, we report that QDot^®^s do not interfere with the biological activity of BMP-2, demonstrating the effectiveness of this probe. To ensure successful attachment of the BMP-2 amino groups to the QDot^®^s carboxyl groups, we used DCC, which is a well-established coupling reagent [54]. Followed by coupling, we utilized size exclusion chromatography (SEC) to separate the BMP-2-QDot^®^s conjugation from other solutes present in the solution. However, SEC limits the volume of the conjugation solution, providing us with only 100 μL. Due to this small volume, large-scale experimentation and multiple repetitions during the same trials were limited. Followed by SEC, we utilized UV/VIS and FTIR spectroscopy to verify BMP-2 was conjugated to QDot^®^s and determine its stability over time [55]. Our data demonstrate that the BMP-2 analog is the most stable to date, as the conjugation remains stable for at least 14 days (Figure 1, Figure 2 and Figure 3). In addition, these findings suggest that it is possible that other BMPs, such as BMP-4 and BMP-6, can be conjugated to QDot^®^s and may be utilized in other research fields.

After observing the successful conjugation of BMP-2-QDot^®^s and determining its stability, we conducted immunofluorescent labeling of C2C12 cells to study the colocalization of BMP-2-QDot^®^s with one of BMP-2’s receptors, BMPRIa. For the first time, our data demonstrate that the BMP-2 analog colocalizes with BMPRIa after one hour of stimulation. Furthermore, some of the BMP-2-QDot^®^s conjugates were internalized into the cell, similar to results published in previous studies [1]. However, shorter and longer stimulation times are needed to determine how quickly BMP-2-QDot^®^s colocalize with BMPRIa and when the complex is internalized into cells. Additionally, subsequent studies are needed to determine how long BMP-2-QDot^®^s remain in cells, if the complex with BMPRIa is shuttled back to the cell surface, or if the complex is degraded [56]. 

We observed the functional activity of the BMP-2-QDot^®^s by using the von Kossa assay. The von Kossa assay uses silver nitrate to bind to phosphate groups, which are produced after pyrophosphate is cleaved by alkaline phosphatase (ALP), a protein that is upregulated by BMP-2 [57,58]. Thus, with BMP-2 present, more ALP should be active, creating more phosphate groups that can be bound to by silver nitrate. With silver nitrate attached to the phosphate groups, the samples are then exposed to UV light, causing mineralization areas to appear as dark deposits. Our data show that the BMP-2-QDot^®^s produce similar mineralization deposits to BMP-2 only stimulation, suggesting that our BMP-2 analog is functional. Furthermore, both the BMP-2-QDot^®^s and BMP-2 had significantly higher normalized mineralization when compared to unstimulated cells, which were 4.10 and 4.70, respectively. 

In conclusion, our main goal was to develop a stable and active BMP-2 analog that could be visualized in a cellular context. Although most fluorophores rapidly degrade and are not photostable, QDot^®^s can be used because of its photostability and intense fluorescence [9,10]. QDot^®^s have also been used in previous studies to observe tumor progression and in vivo visualization [2,6]. A concern of using QDot^®^s is that its cadmium and selenide metal core may be toxic in a cellular environment, especially in long-term experimentation [11,59,60,61]. However, previous studies and the current study demonstrate that short-term usage of these QDot^®^s is non-toxic to cells [11,59]. In addition, this conjugation has allowed us to determine the colocalization of BMP-2 with BMPRIa while retaining the biological activity of BMP-2. These results indicate that the BMP-2-QDot^®^s conjugation can be used to observe BMP-2’s activity, allowing future studies to determine aberrant BMP-2 signaling in diseases such as osteoporosis and osteosarcoma. In addition, although studies measure phosphorylation of Smads to determine BMP-2 activity, here, we use the von Kossa assay to measure bone mineralization as the readout of BMP-2 signaling, as multiple other pathways may be involved, such as fibroblast growth factor (FGF) and Wnt signaling [62,63]. The von Kossa assay measures calcium produced after osteogenesis and as demonstrated by equal cell counts in each condition and the colocalization of BMP-2-QDot^®^s and BMPRIa, we confirm that our conjugate is indeed functional (Figure 4 and Figure 5). The von Kossa assay can detect more mineralization due to cell death, however, this is unlikely as the cell number is similar in each condition (Figure 5). To confirm the biological activity of the BMP-2-Qdot^®^ analog, future studies should investigate downstream signaling pathways, such as activation of regulatory Smads and osteogenic genes via Western blotting and polymerase chain reaction (PCR).
